# Use and optimization of different sources of information for genomic prediction

**DOI:** 10.1186/s12711-017-0365-7

**Published:** 2017-12-11

**Authors:** Joanna J. Ilska, Theo H. E. Meuwissen, Andreas Kranis, John A. Woolliams

**Affiliations:** 1 Roslin Institute (Edinburgh), Easter Bush, Midlothian, EH25 9RG UK; 2 Department of Animal and Aquacultural Sciences, Norwegian University of Life Sciences, Ås, Norway; 3 Aviagen Ltd., 11 Lochend Road Newbridge, Edinburgh, UK

## Abstract

**Background:**

Molecular data is now commonly used to predict breeding values (BV). Various methods to calculate genomic relationship matrices (GRM) have been developed, with some studies proposing regression of coefficients back to the reference matrix of pedigree-based relationship coefficients (**A**). The objective was to compare the utility of two GRM: a matrix based on linkage analysis (LA) and anchored to the pedigree, i.e. ${\mathbf{G}}_{{{\mathbf{LA}}}} ,$ and a matrix based on linkage disequilibrium (LD), i.e. ${\mathbf{G}}_{{{\mathbf{LD}}}}$, using genomic and phenotypic data collected on 5416 broiler chickens. Furthermore, the effects of regressing the coefficients of ${\mathbf{G}}_{{{\mathbf{LD}}}}$ back to **A** (LDA) and to ${\mathbf{G}}_{{{\mathbf{LA}}}}$ (LDLA) were evaluated, using a range of weighting factors. The performance of the matrices and their composite products was assessed by the fit of the models to the data, and the empirical accuracy and bias of the BV that they predicted. The sensitivity to marker choice was examined by using two chips of equal density but including different single nucleotide polymorphisms (SNPs).

**Results:**

The likelihood of models using GRM and composite matrices exceeded the likelihood of models based on pedigree alone and was highest with intermediate weighting factors for both the LDA and LDLA approaches. For these data, empirical accuracies were not strongly affected by the weighting factors, although they were highest when different sources of information were combined. The optimum weighting factors depended on the type of matrices used, as well as on the choice of SNPs from which the GRM were constructed. Prediction bias was strongly affected by the chip used and less by the form of the GRM.

**Conclusions:**

Our findings provide an empirical comparison of the efficacy of pedigree and genomic predictions in broiler chickens and examine the effects of fitting GRM with coefficients regressed back to a reference anchored to the pedigree, either **A** or ${\mathbf{G}}_{{{\mathbf{LA}}}}$. For the analysed dataset, the best results were obtained when ${\mathbf{G}}_{{{\mathbf{LD}}}}$ was combined with relationships in **A** or ${\mathbf{G}}_{{{\mathbf{LA}}}}$, with optimum weighting factors that depended on the choice of SNPs used. The optimum weighting factor for broiler body weight differed from weighting factors that were based on the density of SNPs and theoretically derived using generalised assumptions.

## Background

Thanks to recent advances in genomic technologies, increasing amounts of genotypes are generated worldwide for many livestock species. A central use in animal breeding is the prediction of estimated breeding values (EBV). As genomic data accumulate, these estimates are expected to become more accurate than those obtained using traditional methods based on best linear unbiased predictions (BLUP) [1] that use phenotype and pedigree information only [2].

In the pedigree-based BLUP methodology, the genetic (co)variances of the breeding values (BV) of individuals in the population are modelled by the numerator relationship matrix (${\mathbf{A}}$) scaled by the additive genetic variance. For genomic predictions, it is common to infer genomic relationships by using information on linkage disequilibrium (LD) from the identity-by-state (IBS) among individuals at marker loci [3]. The matrix of these observed relationships (${\mathbf{G}}$) offers more informed estimates of relationships among individuals than pedigree alone, with the added benefit of accounting for the different Mendelian sampling among siblings. To obtain genomic EBV, the expected relationship values of ${\mathbf{A}}$ are replaced by those of ${\mathbf{G}}$ in the mixed model equations, which is referred to as genomic BLUP (GBLUP).

Underlying GBLUP is the idea of an equivalent ridge regression model on the allele counts to exploit LD between markers and causative quantitative trait loci (QTL). A genomic relationship matrix that is fully based on LD (${\mathbf{G}}_{{{\mathbf{LD}}}}$) removes the assumption of an unrelated base population that is made when constructing ${\mathbf{A}}$ and implicitly traces relationships that precede those contained in the pedigree [4], and makes it feasible to obtain EBV without knowledge of the pedigree. However, unless the dataset is very large, the accuracy of the EBV obtained with the LD approach can deteriorate over relatively few generations, since LD is broken down by recombination during meiosis [5]. Since the underlying methodology of the LD approach is based on the association between markers and phenotypes, the choice of SNPs used as markers and their location may influence the results and efficacy of this approach.

A drawback of the LD-based approach is the imperfect linkage between markers and QTL, which can result in over-estimation of marker effects and sampling errors in the genomic relationship coefficients [6]. As such, it has been proposed that bias in relationship estimates may be alleviated by regressing the relationship coefficients of ${\mathbf{G}}$ towards the reference values in ${\mathbf{A}}$. VanRaden [3] proposed a deterministic way of deriving the optimum regression coefficient based on the number of markers available and suggested that, given a large enough number of markers, the optimum regression coefficient may be as large as 0.95, which represents only a small change to the values of ${\mathbf{G}}$.

Irrespective of their indirect effect on the trait, markers can provide invaluable information on the inheritance of chromosome segments, tracked from the base population down the pedigree, which can be used to form an identity-by-descent (IBD) matrix [7]. A linkage analysis (LA) approach combines the theoretical assumptions of ${\mathbf{A}}$ that individuals in the base population are unrelated with observed sharing of marker alleles among genotyped individuals. Therefore, a genomic relationship matrix constructed by using the LA-based approach (${\mathbf{G}}_{{{\mathbf{LA}}}}$) has a structure that is defined by families and assumes that genetic variants that are present in the base population are distinct, in spite of being IBS. Since the method uses markers to track recombinations in the genome, rather than associations with phenotypes, the choice of SNPs may not influence predictions from the LA approach to the same degree as those from the LD approach.

From the assumptions of these three approaches (pedigree, LD, and LA), it follows that the relationships among individuals in the base generation of the pedigree are the same for the pedigree- and LA-based approaches, while they have values that are estimated directly from the genotype data in the LD approach. Since each of these methods represents a different source of information for predicting EBV, a flexible approach that combines their contributions could provide for optimal use of genotypes and pedigree. Therefore, the objective of this study was to evaluate the performance of the ${\mathbf{A}}$, ${\mathbf{G}}_{{{\mathbf{LD}}}}$ and ${\mathbf{G}}_{{{\mathbf{LA}}}}$ matrices, as well as their composites, when fitted to (G)BLUP models for EBV of broiler chickens. The fit of the models to data was assessed by their likelihood, while the efficacy of predicting BV of selection candidates was evaluated using empirical accuracy and bias estimates. To assess the possible effect of the choice of SNPs on the performance of the tested methods, matrices were calculated on two different in silico chips.

## Methods

### Data

The dataset used in the analysis was provided by Aviagen Ltd and consisted of data on 5416 broiler chickens, 1089 males and 4327 females, over six generations. All animals came from a commercial pedigreed female-parent line of broiler breeders that had been closed for 30 years. As described elsewhere [8], the breeding objective was broadly defined and balanced across growth, efficiency, reproductive performance, welfare and health-related traits. A detailed description of the housing and husbandry conditions under which these animals were reared is in [9]. The pedigree had a base population of 288 individuals and a total of 320 sires and 1132 dams, with an average number of offspring of 16 and 5 per sire and dam, respectively. The animals were assigned to contemporary groups of 193 hatch weeks (HW), with on average 26 individuals per HW. Of these, 1446 animals (sires and grand-sires) were genotyped at high-density using the Affymetrix Axiom 600k chip [10], while their offspring were genotyped at low-density (3k Illumina chip [11]) and imputed up to the 600k chip using AlphaImpute [12]. The accuracy of imputation was validated independently of this study, and was found to be greater than 0.97 (unpublished data). The phenotype used was juvenile body weight (BWT), which was recorded at 35 days of age on both sexes on all animals.

The population was split into a training population (TRN) and testing population (TST), consisting of 3146 and 2270 individuals, respectively. The TST individuals were offspring and siblings of individuals in the TRN and none of them had offspring with records included in the TRN. Phenotypes of TST individuals were masked when estimating variances and predicting breeding values and were later used to evaluate empirical accuracy and bias of predictions.

### Quality control and choice of SNPs

The genotypes for the Affymetrix chip were assessed using quality control (QC) procedures within PLINK [13]. After QC, 431k SNPs (69% of total) with known chromosomal locations (based on the chicken genome assembly version 4, i.e. GalGal4) remained and were distributed across 27 chromosomes, including all macro-chromosomes (chromosomes 1 to 5), intermediate chromosomes (6 to 10), and 17 of the 28 micro-chromosomes. Table 1 summarises the SNPs that failed particular screening criteria.

**Table 1:** Quality control criteria and number of markers failing each criterion expressed as a percentage of the total 625,995 SNPs

Category	Quality criterion	Proportion of rejected SNPs
Hardy–Weinberg equilibrium	*P* ≤ 0.001	3.6%
Completeness among individuals	≤ 0.95	5.0%
Minor allele frequency	< 0.01	25.0%
Remaining SNPs		431,249 (69% of all SNPs)

Some markers failed more than one of the quality criteria

From the 431k SNPs that passed QC, two in-silico chips were created, each with ~ 27k SNPs (1000 per chromosome): (1) a panel with near evenly spaced markers, i.e. the ESM chip, and (2) a panel with markers selected for their effect on the trait derived using genome-wide association (GWA), i.e. the GWAM chip, as described below. Since the number of SNPs per chromosome was kept constant in spite of large differences in map length, the density of SNPs on the micro-chromosomes was higher than on macro- and intermediate chromosomes.

#### ESM chip

SNPs on each chromosome were selected according to their linkage map spacing. The linkage map used was assembled from the accumulated Aviagen data. When multiple SNPs were available, those with a high minor allele frequency (MAF) were preferred. For micro-chromosomes 25, 26, 27 and 28, the number of SNPs selected was 888, 998, 998 and 991 respectively, resulting in 26,875 SNPs.

#### GWAM chip

SNPs were selected based on a GWA analysis of BWT conducted using only the TRN set, and carried out in PLINK [13]. SNPs were ranked for each chromosome according to their *P* value. The top 1000 SNPs on each of the 27 chromosomes were selected (resulting in 27,000 SNPs on the chip), irrespective of the threshold for genome-wide significance.

**Fig. 1:**
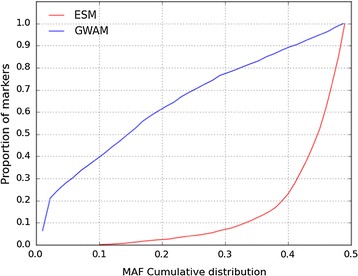
Cumulative distribution of MAF for the ESM and GWAM chips

**Fig. 2:**
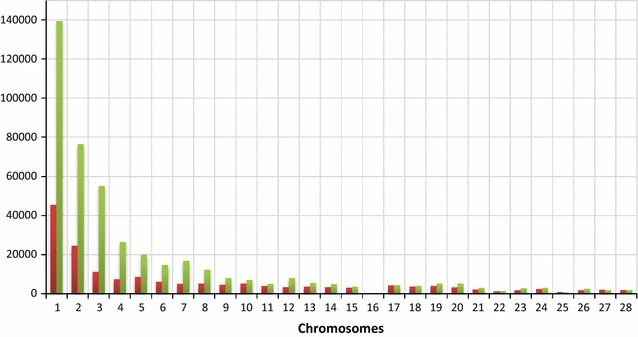
Median distance in base pairs between SNPs on the ESM (green) and GWAM (red) chips

These two chips differed in the average MAF of the SNPs selected, as shown on Fig. 1, with the ESM chip favouring SNPs with a higher MAF and the GWAM chip favouring SNPs with a lower MAF. The distribution of the inter-marker intervals also differed between chips (Fig. 2).

### Calculation of relationship matrices

Different relationship matrices were calculated for individuals in the total population (TST plus TRN). The numerator relationship matrix ${\mathbf{A}}$ was calculated using ASReml procedures [14]. A relationship matrix constructed using linkage analysis (${\mathbf{G}}_{{{\mathbf{LA}}}}$) was calculated using the linkage disequilibrium multi-locus iterative peeling method (LDMIP) described by Meuwissen et al. [7], with the elements of ${\mathbf{G}}_{{{\mathbf{LA}}}}$ obtained by averaging the relationship calculated for each locus. A relationship matrix based on linkage disequilibrium (${\mathbf{G}}_{{{\mathbf{LD}}}}$) was constructed using the ACTA software package [15] following Method 2 of VanRaden [3].

#### Composite relationship matrices

As each of the above matrices uses a different source of information, integrating such information may maximize the benefit of using SNP genotypes [3, 6, 14]. Integration of relationships was done by weighting the relationship coefficients from two matrices ${\mathbf{M}}_{1}$ and ${\mathbf{M}}_{2}$ according to:

$${\mathbf{M}} =\uplambda{\mathbf{M}}_{1} + \left( {1 -\uplambda} \right){\mathbf{M}}_{2} .$$

Two types of integration were considered: LDA, where ${\mathbf{M}}_{1} = {\mathbf{G}}_{{{\mathbf{LD}}}}$ and ${\mathbf{M}}_{2} = {\mathbf{A}}$, and LDLA where ${\mathbf{M}}_{1} = {\mathbf{G}}_{{{\mathbf{LD}}}}$ and ${\mathbf{M}}_{2} = {\mathbf{G}}_{{{\mathbf{LA}}}}$. The optimum weighting factor was found by incrementing $\uplambda$ from 0 to 1, in 0.1 steps. Options with $\uplambda = 1$ always represented information obtained only from ${\mathbf{G}}_{{{\mathbf{LD}}}}$, while $\uplambda = 0$ sourced all information from ${\mathbf{A}}$ in LDA or from ${\mathbf{G}}_{{{\mathbf{LA}}}}$ in LDLA.

### Prediction of breeding values

Linear mixed models were fitted to the TRN data using all relationship matrices described above: 11 in the LDA sequence and 11 in the LDLA sequence, with ${\mathbf{G}}_{{{\mathbf{LD}}}}$ common to both sequences. The mixed linear models (MLM) were fitted using ASReml [12] as follows:

$${\mathbf{y}} = {\mathbf{X}}{\varvec{\uptau}} + {\mathbf{Zu}} + {\mathbf{e}},$$

where ${\mathbf{y}}$ denotes the vector of observations, ${\varvec{\uptau}}$ the vector of estimates for the fixed effects of hatch week and sex, with the design matrix ${\mathbf{X}}$; ${\mathbf{u}}$ is the vector of breeding values with the design matrix ${\mathbf{Z}}$; and ${\mathbf{e}}$ is the vector of residual environmental effects. The breeding values ${\mathbf{u}}$ were assumed to be random and distributed as $MVN\left( {0,V_{A} {\mathbf{M}}} \right)$, where $V_{A}$ is the additive genetic variance and ${\mathbf{M}}$ is a relationship matrix as described above. The residual effects were assumed to be random and distributed $MVN\left( {0,V_{E} {\mathbf{I}}} \right)$, where $V_{E}$ is the residual variance and ${\mathbf{I}}$ is an identity matrix.

Log-likelihoods were used to compare the fit of the models to the TRN data through the log-likelihood ratio test. The log-likelihood profile was calculated for both LDA and LDLA as a function of $\uplambda$. From these profiles, the value of $\uplambda$ at the peak was obtained, together with the corresponding 95% support intervals. The latter were calculated as the interval for which twice the drop in log-likelihood from the peak value was less than 3.84, i.e. twice the difference in log-likelihood was smaller than the critical value of a chi-squared distribution with 1 degree of freedom.

### Empirical accuracy and bias

The practical application of genomic evaluation depends on the empirical accuracy of BV prediction and its bias. These were assessed using the TST set, with phenotypic records masked in BV prediction and revealed for the calculation of empirical accuracy and bias. The empirical accuracy was calculated from the residual correlations of the predicted breeding values (EBV) with the phenotypes, after fitting a linear model to both EBV and phenotype to account for the fixed effects of hatch week and sex. This linear model was fitted separately to the EBV and phenotype in the TST data only, using the GenStat software [16]. To approximate the empirical accuracy of prediction for the breeding value, the residual correlation was divided by the square root of the estimate of heritability ($h^{2}$) for BWT. The same value of $h^{2}$ = 0.35 was assumed throughout and was obtained by using ${\mathbf{A}}$ with the TRN set. This estimate is consistent with published estimates for BWT [17].

The bias was estimated by the regression coefficient of BWT in the TST set on EBV in a fixed linear model that included the fixed effects of sex and hatch week. A regression coefficient of 1 is consistent with no bias, since a difference in EBV between two individuals is an unbiased prediction of the difference in their true BV, and consequently in their phenotypes (given the basic assumption that the phenotype is the sum of the BV and other terms independent of the BV). Regression coefficients greater or less than 1 are indicative of under- and over-prediction of differences in BV, respectively.

## Results

Although the two types of chips used for creating the relationship matrices between individuals consisted of approximately the same number of SNPs, the choice of the SNPs changed the profile of the likelihood for models fitted to the data. To facilitate presentation of results, the findings from analyses carried out using the ESM chip are presented first, followed by results obtained from analyses using the GWAM chip. The comparison between the chips is provided at the end of this section.

### ESM chip

#### Model likelihoods

**Fig. 3:**
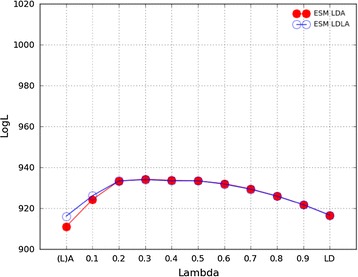
Log-likelihood profile of LDA and LDLA models when using the ESM chip for different ${\varvec{\uplambda}}$ values

Figure 3 presents the likelihood profile of LDA and LDLA analyses with different λ values based on the ESM chip data. The profile of the likelihood was similar for LDA and LDLA, with a relatively flat, convex curve. The likelihood increased gradually from $\uplambda = 0$ to the maximum reached at $\uplambda = 0.3$, and then steadily decreased as $\uplambda$ increased to $1.0$. The largest difference in the likelihood profiles for LDA and LDLA methods was observed at $\uplambda = 0.0$, where the log-likelihood (logL) for the model with ${\mathbf{G}}_{{{\mathbf{LA}}}}$ alone was higher than that for ${\mathbf{A}}$ alone. However, the magnitude of the difference in logL was small, i.e. 916.1 to 911.0. The logL when fitting ${\mathbf{G}}_{{{\mathbf{LD}}}}$ alone (916.6) was very similar to that when fitting ${\mathbf{G}}_{{{\mathbf{LA}}}}$ alone. The 95% support interval for the maximum likelihood was between $\uplambda = 0.2$ and 0.5 for both LDA and LDLA.

Table 2 presents the estimates of the variance components obtained by using REML based on the ESM data with variable weighting in LDA and LDLA. The profile of changes in $V_{A}$ estimates was similar between methods, with initial peaks at $\uplambda = 0.1$ and $\uplambda = 0.2$, followed by gradual decreases in values towards the smallest estimates at $\uplambda = 1.0$. Estimates of $V_{A}$ decreased from using ${\mathbf{A}}$ to using ${\mathbf{G}}_{{{\mathbf{LA}}}}$, with the smallest estimate found for ${\mathbf{G}}_{{{\mathbf{LD}}}}$. Correspondingly, the smallest estimates of $V_{E}$ were observed at $\uplambda = 0.2$ for both LDA and LDLA at 99.7 and 103.5, respectively. As $\uplambda$ increased, the estimates of $V_{E}$ reached their maximum at $\uplambda = 1.0$, i.e. 119.8. While the pattern of changes in parameter estimates was the same for both methods, estimates obtained with LDLA were marginally less sensitive to changes in $\uplambda$ than those obtained with LDA. Estimates of the phenotypic variance were relatively stable across the range of $\uplambda$ for both methods, ranging between 159.5 at $\uplambda = 0.0$ to 161.8 at $\uplambda = 0.8$ for LDA, and from 159.8 at $\uplambda = 0.0$ to 161.7 at $\uplambda = 0.8$ for LDLA. The estimate of heritability ($h^{2}$) was highest when $\uplambda = 0.1$ for both methods, i.e. 0.38 and 0.35 for LDA and LDLA, respectively; however, these differences were small compared to their standard errors.

**Table 2:** Estimates of heritability and variance components based on the ESM chip and obtained from REML using LDA and LDLA composite relationship matrices

$\uplambda$	LDA			LDLA		
	$V_{A}$ (SE)	$V_{E}$ (SE)	$h^{2}$ (SE)	$V_{A}$ (SE)	$V_{E}$ (SE)	$h^{2}$ (SE)
0.0	54.94 (7.41)	104.54 (5.20)	0.34 (0.04)	53.49 (6.77)	106.28 (4.72)	0.33 (0.04)
0.1	61.02 (7.61)	99.72 (5.13)	0.38 (0.04)	56.58 (6.81)	103.60 (4.63)	0.35 (0.04)
0.2	61.02 (7.41)	99.69 (4.94)	0.38 (0.04)	56.59 (6.70)	103.52 (4.52)	0.35 (0.03)
0.3	59.72 (7.19)	100.99 (4.75)	0.37 (0.04)	55.85 (6.59)	104.32 (4.42)	0.35 (0.03)
0.4	57.98 (6.98)	102.89 (4.57)	0.36 (0.04)	54.79 (6.49)	105.61 (4.31)	0.34 (0.03)
0.5	55.99 (6.77)	105.14 (4.40)	0.35 (0.03)	53.51 (6.39)	107.24 (4.21)	0.33 (0.03)
0.6	53.80 (6.56)	107.64 (4.24)	0.33 (0.03)	51.98 (6.27)	109.17 (4.11)	0.32 (0.03)
0.7	51.33 (6.33)	110.36 (4.09)	0.32 (0.03)	50.11 (6.13)	111.38 (4.01)	0.31 (0.03)
0.8	48.50 (6.06)	113.31 (3.96)	0.30 (0.03)	47.79 (5.95)	113.89 (3.92)	0.30 (0.03)
0.9	45.18 (5.75)	116.47 (3.84)	0.28 (0.03)	44.88 (5.70)	116.72 (3.83)	0.28 (0.03)
1.0 (LD)	41.24 (5.36)	119.84 (3.75)	0.26 (0.03)	41.24 (5.36)	119.84 (3.75)	0.26 (0.03)

*h* ^2^, heritability; *V*_*A*_, additive genetic variance; *V*_*E*_, error variance; SE, standard errors in brackets

#### Empirical accuracy and bias

**Fig. 4:**
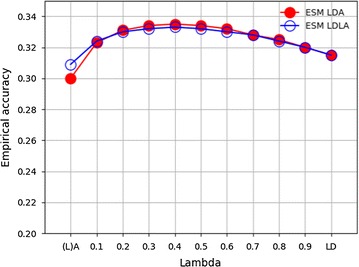
Residual empirical accuracy of BV predictions using LDA and LDLA with the ESM chip. Filled circles indicate empirical accuracy of LDA BV prediction, empty circles indicate empirical accuracy of LDLA BV prediction

Figure 4 presents empirical accuracies of BV prediction obtained from LDA and LDLA based on the ESM chip. Given the size of the training population, the greatest empirical accuracy was achieved when marker information was supported by pedigree structure, i.e. by using either ${\mathbf{A}}$ or ${\mathbf{G}}_{{{\mathbf{LA}}}}$, at $\uplambda = 0.4$. The benefit from including pedigree information was marginally higher for LDA, which resulted in a greater empirical accuracy than LDLA for 0.2 ≤ $\uplambda$ ≤ 0.6. Overall, the changes in empirical accuracy that were observed were small, with values of 0.30 when using ${\mathbf{A}}$ alone and of 0.31 when genomic information was used.

Table 3 shows the bias of predictions, presented as regression coefficients obtained from regressing phenotypes of individuals in the TST population on their EBV obtained from LDA and LDLA analyses, using both chips. For the ESM chip, using ${\mathbf{G}}_{{{\mathbf{LA}}}}$ at $\uplambda = 0.0$ resulted in marginally less bias than using ${\mathbf{A}}$, however the differences between methods were small compared to their standard errors for both LDA and LDLA. Overall, differences in the EBV overestimated differences in phenotypes and, by inference, differences in true BV, across the methods and range of weighting factors used. A bias in EBV is a common result in populations that are under selection, when, as was the case here, the multi-stage, multi-trait and multi-generational selection criteria that were used cannot be easily accommodated in a model.

**Table 3:** Estimates of bias when using the ESM and GWAM chip obtained from regressing phenotypes for the TST population on BV predicted using LDA and LDLA composite relationship matrices using different $\uplambda$ values

	ESM		GWAM	
	LDA	LDLA	LDA	LDLA
$\uplambda = 0$	0.71 (0.08)	0.73 (0.08)	0.71 (0.08)	0.77 (0.08)
$\uplambda = 0.1$	0.71 (0.08)	0.72 (0.08)	0.62 (0.07)	0.67 (0.07)
$\uplambda = 0.2$	0.71 (0.08)	0.71 (0.08)	0.58 (0.07)	0.63 (0.07)
$\uplambda = 0.3$	0.70 (0.07)	0.71 (0.08)	0.56 (0.07)	0.60 (0.07)
$\uplambda = 0.4$	0.70 (0.07)	0.70 (0.07)	0.54 (0.07)	0.58 (0.07)
$\uplambda = 0.5$	0.69 (0.07)	0.69 (0.07)	0.52 (0.07)	0.56 (0.07)
$\uplambda = 0.6$	0.68 (0.07)	0.69 (0.07)	0.51 (0.07)	0.53 (0.07)
$\uplambda = 0.7$	0.68 (0.07)	0.68 (0.07)	0.49 (0.07)	0.51 (0.07)
$\uplambda = 0.8$	0.68 (0.07)	0.68 (0.07)	0.47 (0.07)	0.49 (0.07)
$\uplambda = 0.9$	0.68 (0.07)	0.68 (0.07)	0.46 (0.07)	0.46 (0.07)
$\uplambda = 1$	0.68 (0.08)	0.68 (0.07)	0.44 (0.07)	0.44 (0.07)

Standard errors in brackets

### GWAM chip

#### Model likelihoods

**Fig. 5:**
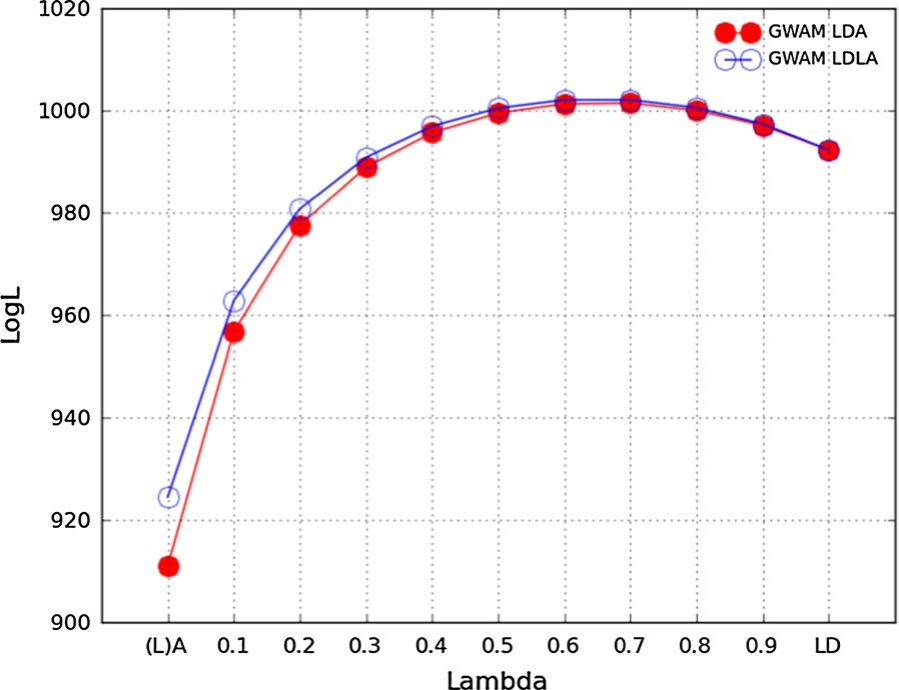
The log-likelihood profile of LDA and LDLA models when using the GWAM chip for different $\uplambda$ values

Figure 5 presents the likelihood profile of LDA and LDLA analyses with different $\uplambda$ values based on the GWAM chip. For both LDA and LDLA, the likelihood profile was similar across values of $\uplambda$, except for small $\uplambda$, for which LDLA (using ${\mathbf{G}}_{{{\mathbf{LA}}}}$) showed a greater likelihood than LDA (using ${\mathbf{A}}$). The logL with ${\mathbf{G}}_{{{\mathbf{LA}}}}$ alone was greater than with ${\mathbf{A}}$ alone, at 924.5 and 911.0, respectively. The change in logL was rapid for small $\uplambda$ (0.0 < λ < 0.3) but slowed down for λ > 0.4. The maximum logL was reached at $\uplambda = 0.7$ for both LDA and LDLA, with the 95% support interval for $\uplambda$ between 0.5 and 0.8 (inclusive).

Table 4 presents estimates of the variance components obtained through REML run on the data using SNPs from the GWAM chip, with both LDA and LDLA. Most of the differences between estimates were not statistically significant (*P* > 0.05), with the exception of the difference between the highest and lowest heritability estimates in LDA.

**Table 4:** Estimates of heritability and variance components from LDA and LDLA analyses when using the GWAM chip with different composite relationship matrices

	LDA			LDLA		
	$V_{A}$	$V_{E}$	$h^{2}$	$V_{A}$	$V_{E}$	*h* ^2^
$\uplambda = 0$	54.94 (7.41)	104.54 (5.21)	0.34 (0.04)	57.26 (7.08)	103.78 (4.74)	0.36 (0.04)
$\uplambda = 0.1$	68.43 (7.80)	92.25 (5.05)	0.43 (0.04)	64.25 (7.10)	96.25 (4.53)	0.40 (0.04)
$\uplambda = 0.2$	66.53 (7.44)	92.06 (4.79)	0.42 (0.04)	62.13 (6.79)	96.13 (4.36)	0.39 (0.03)
$\uplambda = 0.3$	63.53 (7.12)	93.59 (4.57)	0.40 (0.04)	59.61 (6.55)	97.14 (4.22)	0.38 (0.03)
$\uplambda = 0.4$	60.60 (6.83)	95.62 (4.38)	0.39 (0.04)	57.31 (6.35)	98.54 (4.11)	0.37 (0.03)
$\uplambda = 0.5$	57.84 (6.58)	97.88 (4.20)	0.37 (0.03)	55.23 (6.19)	100.15 (4.00)	0.36 (0.03)
$\uplambda = 0.6$	55.23 (6.34)	100.27 (4.05)	0.36 (0.03)	53.28 (6.05)	101.93 (3.90)	0.34 (0.03)
$\uplambda = 0.7$	52.66 (6.11)	102.78 (3.90)	0.34 (0.03)	51.34 (5.91)	103.89 (3.81)	0.33 (0.03)
$\uplambda = 0.8$	49.99 (5.88)	105.46 (3.77)	0.32 (0.03)	49.22 (5.76)	106.09 (3.72)	0.32 (0.03)
$\uplambda = 0.9$	46.94 (5.62)	108.39 (3.66)	0.30 (0.03)	46.64 (5.57)	108.64 (3.64)	0.30 (0.03)
$\uplambda = 1$ (LD)	43.08 (5.28)	111.73 (3.57)	0.28 (0.03)	43.08 (5.28)	111.73 (3.57)	0.28 (0.03)

*h* ^2^, heritability; *V*_*A*_, additive genetic variance; *V*_*E*_, error variance; SE, standard errors in brackets

Models using ${\mathbf{A}}$ and ${\mathbf{G}}_{{{\mathbf{LA}}}}$ gave similar variance component estimates; both had greater estimates of $V_{A}$ and smaller estimates of $V_{E}$ than those obtained using ${\mathbf{G}}_{{{\mathbf{LD}}}}$, with similar estimates of $h^{2}$ for ${\mathbf{A}}$ and ${\mathbf{G}}_{{{\mathbf{LA}}}}$ (0.34 and 0.36, respectively; SE = 0.04), and a lower estimate of $h^{2}$ of 0.28 (SE = 0.03) for ${\mathbf{G}}_{{{\mathbf{LD}}}}$.

For both LDA and LDLA, there was a consistent pattern in the change of the variance estimates with increasing values of $\uplambda$; the estimates of $V_{A}$ were greatest at $\uplambda = 0.1$ when pedigree information was supported by ${\mathbf{G}}_{{{\mathbf{LD}}}}$ relationships, and gradually decreased with increasing $\uplambda$ to the smallest value when only ${\mathbf{G}}_{{{\mathbf{LD}}}}$ information was used. This pattern was mirrored for estimates of $V_{E}$, which were smallest at $\uplambda = 0.2$ and gradually increased with $\uplambda$. Estimates of $h^{2}$ obtained in LDLA for λ > 0 were lower than in equivalent LDA models, however, these differences were mostly small.

#### Empirical accuracy and bias

**Fig. 6:**
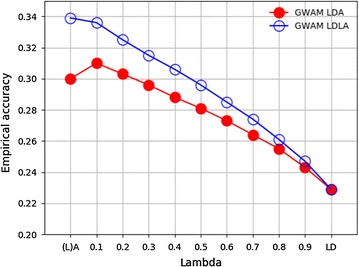
Residual empirical accuracy of BV predictions using LDA and LDLA with the GWAM chip. Filled circles indicate empirical accuracy of LDA BV prediction, empty circles indicate empirical accuracy of LDLA BV prediction

Figure 6 presents empirical accuracies of BV predictions obtained from LDA and LDLA using the GWAM chip. Empirical accuracy was greatest for predictions based on ${\mathbf{G}}_{{{\mathbf{LA}}}}$, i.e. 0.34, and smallest for predictions based on ${\mathbf{G}}_{{{\mathbf{LD}}}}$. For both LDA and LDLA, the change in empirical accuracy with increasing λ was almost linear, particularly for $\uplambda$ between 0.2 and 0.8. For LDA, inclusion of ${\mathbf{G}}_{{{\mathbf{LD}}}}$ information with $\uplambda = 0.1$ improved the empirical accuracy compared to that obtained through fitting ${\mathbf{A}}$ alone.

Table 3 shows the bias of BV predictions obtained from LDA and LDLA analyses using the GWAM chip. The smallest bias was found for predictions using ${\mathbf{G}}_{{{\mathbf{LA}}}}$ only, i.e. 0.77 (SE = 0.08), which represents an overestimate of true differences by EBV. Similarly for LDA, the smallest bias, was observed at $\uplambda = 0$, i.e. using ${\mathbf{A}}$. Generally, the LDLA resulted in less bias than the LDA method across the range of $\uplambda$, although the associated standard errors were relatively large. For both methods, the bias and overestimation increased substantially as more weight was given to ${\mathbf{G}}_{{{\mathbf{LD}}}}$.

### Comparison of chips

The choice of markers had a considerable effect on the results observed. Selection of SNPs through GWA hits resulted in a considerably higher likelihood of ${\mathbf{G}}_{{{\mathbf{LD}}}}$-based analyses than when using the ESM chip, at 992.3 and 916.6, respectively, for $\uplambda = 1.0$. ${\mathbf{G}}_{{{\mathbf{LA}}}}$ was less affected by the choice of SNPs, with logL values of 924.5 and 916.1, for the GWAM and ESM chips, respectively, for $\uplambda = 0$. As a result, the profile of changes in the likelihood with increasing $\uplambda$ was much flatter for the ESM chip over the range of $\uplambda$, since logL when using ${\mathbf{G}}_{{{\mathbf{LD}}}}$ ($\uplambda = 1.0$) and ${\mathbf{G}}_{{{\mathbf{LA}}}}$ ($\uplambda = 0$) were very similar. The confidence interval for the maximum likelihood value contained different values of $\uplambda$ for the two chips, i.e. from 0.2 to 0.5 for the ESM chip, and from 0.5 to 0.8 for the GWAM chip. Estimates of $V_{A}$ based on ${\mathbf{G}}_{{{\mathbf{LD}}}}$ were smaller with the ESM chip than with the GWAM chip, while estimates of $V_{E}$ and total variance were greater. Thus, the estimates of $h^{2}$ were higher for the GWAM chip, although most of the differences were within the range of standard errors.

While the likelihood results indicated that the GWAM chip fitted the data better in the training population, the empirical accuracy and bias indicated that it was not as good for prediction as the ESM chip. For the GWAM chip, the empirical accuracy increased as the proportion of information sourced from ${\mathbf{G}}_{{{\mathbf{LD}}}}$ decreased and reached its highest value when all information was obtained from either the ${\mathbf{A}}$ or the ${\mathbf{G}}_{{{\mathbf{LA}}}}$ matrix. Meanwhile, predictions based on the ESM chip achieved the highest empirical accuracy when different sources of information were combined, although the differences between methods were less obvious than for predictions based on the GWAM chip. Predictions based on ${\mathbf{G}}_{{{\mathbf{LD}}}}$ constructed using the GWAM chip had a larger bias, due to overestimation, than those using ${\mathbf{A}}$ or ${\mathbf{G}}_{{{\mathbf{LA}}}}$, or when using the same methodology with the ESM chip.

## Discussion

The study used real data collected on a commercial broiler population to determine the effect of different sources of information on the design of the SNP chip, the likelihood and fit of linear models using different constructions for relationships, and the empirical accuracy and bias of predictions of BV for BWT for selection candidates. Using more than 3500 chickens in the TRN dataset allowed more accurate predictions using genomics than would be obtained using traditional pedigree-based methods and it is expected that the empirical accuracy of genomic predictions will continue to increase as the numbers of genotyped relatives of the selection candidates increases. The highest likelihood and empirical accuracy in the analysed dataset were reached when the relationships used were intermediate between those that track the pedigree from the base generation in ${\mathbf{A}}$ or ${\mathbf{G}}_{{{\mathbf{LA}}}}$, and relationships that were constructed following VanRaden [3] in ${\mathbf{G}}_{{{\mathbf{LD}}}}$. Using a SNP chip obtained from GWA hits resulted in higher likelihoods but less accurate and more biased predictions than a SNP chip based on equal spacing across the genome. Given the major differences that resulted from the two SNP chips used in our study, the discussion will address the outcomes concerning the form of the relationships used in the models and their likelihood in the context of the equally-spaced ESM chip, before addressing their interactions with the design of the SNP chip.

With evenly-spaced SNPs (ESM chip), the use of relationship matrices ${\mathbf{A}}$, ${\mathbf{G}}_{{{\mathbf{LA}}}}$, and ${\mathbf{G}}_{{{\mathbf{LD}}}}$ showed consistency in their relative likelihoods when fitted to TRN and in accuracies of prediction in TST. The similarity of the empirical accuracies obtained by using ${\mathbf{G}}_{{{\mathbf{LD}}}}$ and ${\mathbf{G}}_{{{\mathbf{LA}}}}$ indicates that, with this magnitude of training set size, very little information was available from relationships that were already present in the base generation of the pedigree, or from associations with large QTL. This result is supported by previous findings which suggested that, for polygenic traits, the effect of historical covariances in BV within the base generation of the pedigree on covariances in BV of more recent generations is limited [5, 18]. Generally, the partitioning of the sources of information in the LD approach depends on the genetic architecture of the trait in question, with traits influenced by large-effect QTL being less affected by family connections, since most of the information is sourced from QTL associations in that case [4]. Body weight in chickens is considered to be a largely polygenic trait, with variance explained by multiple QTL and their epistatic interactions [19], which explains the observed similarity in performance of ${\mathbf{G}}_{{{\mathbf{LA}}}}$ and ${\mathbf{G}}_{{{\mathbf{LD}}}}$. However, this result would not necessarily be repeated for traits with a different architecture.

Combining the LD and LA sources of information increased both the likelihood of the models (for both the GWAM and ESM chips) and the empirical accuracy of predictions. The idea of mixing LD and LA information was first introduced for QTL mapping [2]. Regressing the genomic relationships obtained for ${\mathbf{G}}_{{{\mathbf{LD}}}}$ back to some reference was suggested as a method by VanRaden [3] and subsequently explored by Goddard et al. [6]. Both these publications chose the reference point to be ${\mathbf{A}}$, a pedigree-based expectation, but here the use of ${\mathbf{G}}_{{{\mathbf{LA}}}}$ as a pedigree-based alternative is also explored. The extent of regression was justified by the idea of weighting the two estimates of relationship according to the inverse of their error variances for the true relationships. VanRaden [3] and Goddard et al. [6] offered deterministic predictions for $\uplambda$, both with the form of $\uplambda = m/\left( {m + k} \right)$ where $m$ is the number of markers with some constant *k.* VanRaden [3] suggested $k = 50$ derived from the prediction error variance for the true fraction of DNA shared between full-sibs, whereas Goddard et al. [6] set $k = M_{e}$, where $M_{e}$ is the effective number of independent chromosomal segments.

Several studies have suggested ways of deriving $M_{e}$, with somewhat variable results. For example, calculation of $M_{e}$ using the formula of Meuwissen et al. [20] and the genome map of Groenen et al. [21] resulted in estimates ranging from 584 to 1584, for an effective population size ($N_{e}$) ranging from 50 to 200, where $N_{e}$ estimates were based on the study of broiler populations by Andreescu et al. [22]. In contrast, an empirical estimate of 7800 was obtained if the formula of Daetwyler et al. [23] is inverted based on the achieved accuracy, observed heritability, and assuming the ESM chip captures a 0.8 fraction of the additive genetic variance. Considering the variability in the estimates of $M_{e}$, it follows that the range of possible values for $k$ varies widely.

The optimum weighting factor observed for the broiler BWT data used here was substantially different from the theoretical values, suggesting that $k$ is much greater than expected. The empirically-derived support interval for $\uplambda$ was between 0.2 and 0.5, regardless of whether regression was back to ${\mathbf{G}}_{{{\mathbf{LA}}}}$ or ${\mathbf{A}}$, while the predicted $\uplambda$ with *m* = 27,000 are 0.998 based on VanRaden [3] and 0.775 based on Goddard et al. [6] using the empirical $M_{e}$. Even when it is assumed that the ESM chip captures all genetic variation, resulting in an increase in $M_{e}$ up to 11,133, the theoretical value of $\uplambda$ of 0.729 is still outside the empirical support interval. Considering these discrepancies, it appears that the theoretical considerations are unreliable in predicting the extent of regression required. Instead, empirical estimates, as described here, are required to regress ${\mathbf{G}}_{{{\mathbf{LD}}}}$ to ${\mathbf{G}}_{{{\mathbf{LA}}}}$ or ${\mathbf{A}}$ to obtain the best fit of the models to data. Furthermore, the optimum weighting coefficients are likely data dependent, influenced by the genetic architecture of the traits, as well as data structure. For example, it is expected that if the ${\mathbf{G}}_{{{\mathbf{LD}}}}$ was constructed solely from QTN, all information should be sourced from the QTN genotypes, and thus the optimum $\uplambda$ would be 1. This data dependency implies that the value of $\uplambda$ found here for BWT in broilers cannot be generalized to other traits and species. However, it also demonstrates that the theoretical results of VanRaden [3] and Goddard et al [6] cannot be assumed to be absolute or generally appropriate.

Although the optimum weighting factors reported here are considerably smaller than previously suggested approximations, it is likely that they still underestimate the desired levels of regression to pedigree relationships. The data used here consisted of a large number of genotypes that were imputed using AlphaImpute [12], which uses LD structures to trace haplotype inheritance along the pedigree. As such, the resulting genomic relationship coefficients appearing in ${\mathbf{G}}_{{{\mathbf{LD}}}}$ that are based on imputed genotypes are already weighted towards pedigree.

### Performance of the GWAM chip

The results from using the GWAM chip clearly demonstrate (1) the difference in the roles of the genomic data when used for constructing ${\mathbf{G}}_{{{\mathbf{LD}}}}$ or ${\mathbf{G}}_{{{\mathbf{LA}}}}$, and (2) the distinction between the likelihood and goodness of fit obtained within a dataset and the predictive value of the resulting model parameter estimates beyond the data. When genomic data were used for linkage analysis in ${\mathbf{G}}_{{{\mathbf{LA}}}}$, genomic relationships based on the GWAM chip gave a better fit and improved empirical accuracy more than those based on the ESM chip. When, instead, their allelic counts were used as ridge regression variables to construct ${\mathbf{G}}_{{{\mathbf{LD}}}}$, genomic relationships based on the GWAM chip apparently gave a better fit, but gave less accurate and more biased predictions than those based on the ESM chip. It should be noted that the GWA used for SNP selection was carried out using only TRN, so that the choice was not influenced by the genotypes and phenotypes of TST.

The LA approach uses genomic data to construct indicator variables for inheritance from paternal and maternal alleles, individual by individual, along the pedigree. In this case, the prediction does not rely on the validity of an association between performance and a marker allele across the population. Therefore, one might expect that, provided the SNPs cover the genome with a similar distribution of MAF, the choice of SNPs would make very little difference to the fit and empirical accuracy when constructing ${\mathbf{G}}_{{{\mathbf{LA}}}}$. In our study, we observed that, when using the LA approach, both the fit and the empirical prediction accuracy were slightly improved when using the GWAM chip, although SNPs had a lower MAF than the SNPs on the ESM chip. One explanation, other than chance, may be that the GWAM chip allowed greater emphasis on regions that are enriched in QTL for the trait of interest and on the segregation of haplotypes that contained QTL, as it was unconstrained by considerations of spacing over the genome. This may have resulted in more accurate pedigree tracing in these regions. However, if this is the case, then a similar improvement would not be expected for other traits when using the same SNPs.

For constructing ${\mathbf{G}}_{{{\mathbf{LD}}}}$, the genomic data is framed in an underlying ridge regression model and seeks associations between phenotype and allele counts at a locus. Although both the underlying ridge regression model and GWA estimate these associations, the former fits the regressions simultaneously, while GWA fits them one at a time. The GWA results from TRN may be considered as consisting of three broad classes of SNPs regardless of their statistical significance: (1) markers that mark true QTL, likely due to their close proximity to QTL, and for which estimates are relevant to TST since linkage is substantially retained in TST; (2) markers that mark true QTL for which linkage is not retained in TST; and (3) markers that align with phenotypes within TRN by chance alone. The most significant GWA hits in TRN would be expected to capture many of the SNPs in the first of these classes. However, as selection continues down the list of SNPs, those chosen for inclusion are increasingly enriched by the third category, which will consist of SNPs with no true predictive value but with prediction errors of substantial magnitude.

This dissection of the SNP selection process fully explains the results observed for changing $\uplambda$, moving from ${\mathbf{G}}_{{{\mathbf{LA}}}}$ to ${\mathbf{G}}_{{{\mathbf{LD}}}}$. Prior calibration of SNPs to the TRN dataset resulted in higher likelihoods, reduced estimates of the residual error, $V_{E}$, and correspondingly higher $h^{2}$ estimates compared to the ESM chip, as the SNPs on the GWAM chip explained more of the variance, irrespective of the wider relevance of the selected SNPs beyond the TRN data. Therefore, for prediction beyond TRN, the inclusion of SNPs with substantial effects of relevance only to the TRN data reduced the empirical accuracy of prediction and increased bias as more emphasis was placed on the genomic data. The bias observed was over-prediction, which is expected since some of the variance captured by the GWAM SNPs and used for prediction incorporated into the predictors is only locally relevant to phenotypes within TRN and irrelevant to TST. In contrast, choice of SNPs on the ESM chip made no reference to the TRN data and so was less capable of explaining variance locally within the TRN data, but contained no biases in the prediction equations used for the TST data.

These results do not exclude the judicious use of GWA results in the design of SNP chips but emphasise the need for care in avoiding extrapolating the value of putative QTL. This was also shown by Sanchez-Molano et al. [24] in a study on canine hip-dysplasia, which demonstrated that chips that included very few SNPs resulted in higher accuracy when they contained SNPs selected according to GWA hits compared to randomly selected SNPs. However, accuracy increased further by increasing the number of SNPs up to an asymptote, as also demonstrated by Hayes et al. [25]; selecting these extra SNPs randomly reached this asymptote more quickly than selecting them based on significance in the GWA. These results are consistent with those observed in our study.

## Conclusions

Analysing data on body weight from a commercial broiler population showed that genomic selection using relatively low marker densities can improve the likelihood of models and empirical accuracy of predictions of breeding values compared to using pedigree only. The best results for the analysed dataset were achieved when the relationship matrix combined different sources of information, with ${\mathbf{G}}_{{{\mathbf{LD}}}}$, based on IBS, regressed back to ${\mathbf{G}}_{{{\mathbf{LA}}}}$, where markers were used to provide improved relationships based on the pedigree structure. For broiler body weight, the optimum regression coefficient $\uplambda$ was estimated to be between 0.2 and 0.5 for the ESM chip. These optimal regression coefficients differed from theoretically-derived values, which for an equivalent number of SNPs were speculated to be close to 1. It is expected that the optimum regression coefficients are dependent on the genetic architecture of the trait for the population. The apparent increase in goodness of fit from using SNP chips that are based on significance of GWA hits was accompanied by reduced empirical accuracy and greater bias in predictions through the inclusion of SNPs that are calibrated to local features of the training set but are unrepresentative of the testing set.
